# Effect of betulinic acid administration on TLR-9/NF-κB /IL-18 levels in experimental liver injury

**DOI:** 10.3906/sag-2004-184

**Published:** 2021-06-28

**Authors:** Eda DOKUMACIOĞLU, Hatice İSKENDER, Kübra Asena TERİM KAPAKİN, Güler YENİCE, Behzat MOKTHARE, İsmail BOLAT, Armağan HAYIRLI

**Affiliations:** 1 Department of Nutrition and Dietetics, Faculty of Healthy Sciences, Artvin Çoruh University, Artvin Turkey; 2 Department of Pathology, Faculty of Veterinary Medicine, Atatürk University, Erzurum Turkey; 3 Department of Animal Nutrition and Nutritional Disorders, Faculty of Veterinary Medicine, Atatürk University, Erzurum Turkey

**Keywords:** Acetaminophen, betulinic acid, interleukin-18, nuclear factor kappa B, toll-like receptor-9

## Abstract

**Background/aim:**

Acetaminophen (APAP), used in the composition of thousands of preparations, is the most commonly used analgesic and antipyretic drug. The present study aimed to investigate the potential protective effects of the betulinic acid (BA) treatment through an APAP-induced hepatotoxicity rat model, using inflammatory, biochemical, and histopathological parameters.

**Materials and methods:**

The study consisted of four groups: control group, APAP group, BA group, and APAP+BA group. Experimental studies continued for fifteen days. Serum samples were analysed for glucose, total cholesterol (TChol), triglyceride (TG), low density lipoprotein (LDL), high density lipoprotein (HDL), aspartate amino transferase (AST), malondialdehyde (MDA), toll-like receptor-9 (TLR-9), nuclear factor kappa B (NF-κB), and interleukin-18 (IL-18).

**Results:**

TLR9, IL-18, NF-κB, and MDA levels increased significantly in liver injury groups. These increases considerably decreased by the BA treatment. All groups showed immunopositivity for 8-hydroxy-2’–deoxyguanosine (8-OHdG) and interleukin (IL-1β) in the hepatocytes, inflammatory cells, and epithelial cells of bile ducts.

**Conclusion:**

BA can be used as an effective agent in the prevention and treatment of acute liver diseases due to its inhibitory properties in multiple pathways and its potent antioxidant effects.

## 1. Introduction

Acetaminophen (APAP), an ingredient of thousands of preparations, is the most commonly used class of analgesic and antipyretic drugs worldwide because it is inexpensive and easily accessible [1]. APAP is also one of the most common drugs causing poisoning and a highly preferred agent at high doses in suicide attempts [2]. Overdose APAP causes hepatic necrosis and kidney injury via increasing the production of reactive oxygen species (ROS) and decreasing the activity of antioxidant enzymes [3,4]. The pathophysiology of hepatotoxicity is associated with inflammation, disruption of intracellular ion balance, apoptosis, and mitochondrial dysfunction [5].

Toll-like receptors (TLRs) are transmembrane proteins involved in the defense of the immune system against infections, with significant roles in the production of innate immune responses to many pathogens and activation of the acquired immune response, along with the production of various interleukins and other proinflammatory cytokines [6,7]. Today, TLRs are considered as molecules playing a key role in immune response to infections [8]. The TLR-9 is a member of TLR family that bears a recognition pattern for microbial DNA. TLR-9 is also an important factor in autoimmune diseases, and there is active research into synthetic TLR9 agonists and antagonists that help regulate autoimmune inflammation [9]. Expression of TLR-9 is positively linked to the secretion of proinflammatory cytokines tumor necrosis factor-alpha (TNF-α), interleukin-18 (IL-18) and interleukin-6 (IL-6). The TLR-9 induces inflammation via the nuclear factor kappa B (NF-κB) pathway that initiates pro-inflammatory reactions in the immune responses. The TLR-9 agonists and antagonists may be useful in treatment of a variety of inflammatory conditions [10].

The IL-18 is a proinflammatory cytokine produced by various cells such as macrophages, epithelial cells, activated T lymphocytes, osteoblasts, adrenal cortex cells, and intestinal epithelial cells, and it acts as an important regulator of innate and acquired immune responses [11]. The TLRs and the receptor for IL-18 are required for defence system but, if hyper-activated or not switched off efficiently, can cause tissue damage and inflammatory diseases. Understanding how the checks and balances in the system are integrated to fight infection without the network operating out of control is crucial for the development of improved drugs to treat these diseases in the future [11,12]. The NF-κB family consists of a group of transcription factors playing a role in many physiological and pathological events such as cell growth, apoptosis, immune response, and inflammation. Activated with various intrinsic and extrinsic stimuli, NF-κB regulates transcription of numerous genes and plays an important role in the development of chronic inflammatory diseases by increasing the expression of a range of proteins such as cytokines, chemokines, immunoglobulins, and cell adhesion molecules [13,14]. Activation of NF-κB under oxidative stress has been noticed in a number of inflammatory complications. Factors/agents implicate ROS-induced lipid peroxidation products such as lipid aldehydes in the activation of signaling cascade, eventually activates NF-κB [15].

Throughout the historical development, various plant-derived compounds found in nature are among the most frequently used sources for the treatment of diseases and for the discovery and development of new drugs. Increase in diseases generate interest in alternative medicine along with conventional drug treatment, which includes compounds with a high antioxidant content [16]. Betulinic acid (BA) is a pentacyclic triterpenoid isolated from plants growing in tropical climates and exhibiting significant biological activities. The BA was shown to have antioxidant, anti-inflammatory and anti-hyperlipidemic effects and suppress tumor development via antitumor effects [17,18]. The present study aimed to investigate the potential protective effects of the BA treatment through an APAP-induced hepatotoxicity rat model, using inflammatory, biochemical, and histopathological parameters.

## 2. Materials and methods 

APAP and BA were purchased from Sigma Chemical Co (St. Louis, MO, Germany). Sprague–Dawley male rats (n = 36) weighing 200–250 g at the age of 6–8 weeks were housed in special rooms with ambient temperature of 22 ± 2°C and humidity of 50%–60%, under photoperiod of 12: 12 h light: dark. The animals were given tap water and standard diet ad libitum. At first, the animals were divided randomly into two groups: control vs. APAP administration, and then half of these subgroups was assigned randomly to either remained untreated or treated with BA for 15 days. The BA was administered for treatment via oral gavage at a dose of 25 mg/kg [19]. The APAP was dissolved in hot saline and administered on the last day to produce toxic hepatitis via a single oral gavage at a dose of 1 g/kg [20]. The APAP was administered 24 h after last BA treatment. Intraperitoneally ketamine (80 mg/kg) and xylazine (10 mg/kg) administered rats were sacrificed at the end of the experiment, after that, intracardiac blood samples were taken. Blood samples were centrifuged at 3000 rpm for 10 min to separate the sera. Serum samples were analyzed for glucose, total cholesterol (TChol), triglyceride (TG), low density lipoprotein (LDL), high density lipoprotein (HDL), aspartate amino transferase (AST), malondialdehyde (MDA), TLR-9, NF-κB and IL-18 values.

 Serum MDA levels were determined by the method of Ohkawa et al. [21] based on the measurement of the absorbance of the pink colour complex formed by MDA with thiobarbituric acid at a wavelength of 532 nm. The results were given in nmol/L.

TLR-9, NF-κB ve IL-18 levels were measured by using the enzyme-linked immunoassay (ELISA) method using a rat ELISA kit (Cusabio Technology Llc., Houston, TX 77054, USA) according to the manufacturer’s instruction. In the ELISA method, an antigen must be immobilized on a solid surface and then complexed with an antibody that is linked to an enzyme. Detection is accomplished by assessing the conjugated enzyme activity via incubation with a substrate to produce a measureable product. The most crucial element of the detection strategy is a highly specific antibody–antigen interaction. Absorbances were read at 450 nm in the ELISA reader. The TLR-9, IL-18 results were expressed as pg/mL, whereas the NF-κB result was expressed as ng/mL.

At the end of the study, 8 animals were slaughtered from each subgroup. The liver tissue samples were fixed in 10% buffered formalin and routinely processed for the histological examination by embedding in paraffin wax. The tissue sections were cut 4 μm in thickness and stained by the Haematoxylin-Eosin for observation under a light microscope. They were also evaluated by high–power light microscopic examination using an Olympus Bx51 with a DP72 camera system. Each specimen was examined in 10 randomly selected areas of approximately an X40 objective. The scores were derived semi-quantitatively on the preparations from each rat and were reported as follows: Grade 0 = – (negative); Grade 1 = +1 (mild); Grade 2 = +2 (moderate); Grade 3 = +3 (severe); Grade 4 = +4 (most severe) [22].

Four μm sections from all of the tissue samples were cut and processed for immunohistochemical examination by a standard avidin-biotin-peroxidase method that the producer described. Rabit policlonal antibodies that react with rat 8-OHdG (sc-66036) the dilution of 1:200 and IL-1β antibody (Catalog No. ab9722, the dilution of 1: 200 were used for for 60 min. A secondary antibody was used according to the manufacturer’s protocol (expose mouse and rabbit-specific HRP/DAB detection IHC Kit, Abcam Cat. No. ab80436). After three washes with 0.1% Tween 20 in PBS, the sections were incubated with 3,3-diaminobenzidine (Dako Cytomation) and counterstained with Mayer’s hematoxylin (DakoCytomation, Glostrup, Denmark) [22].

Tissue sections were evaluated by high-power light microscopic examination using an Olympus Bx51 with a DP72 camera system. Each specimen was examined in 10 randomly selected areas of approximately an X40 objective. The scores were derived semi-quantitatively using light microscopy on the preparations from each rat and were reported as follows: Grade 0 = – (negative); Grade 1 = +1 (mild); Grade 2 = +2 (moderate); Grade 3 = +3 (severe); Grade 4 = +4 (most severe) [22].

Two–way ANOVA was used to test the main effect of health status (healthy vs. damaged) and treatment (control vs. betulinic acid) as well as their interaction on continuous variables (blood biochemistry parameters). The linear model was: Yijk= µ + HSi + Trtj + (HS x Trt)ij+ eijk, where Yijk = response variable, µ = population mean, HS = ith health status, Trt = jth treatment, eijk = random error, being equal and normally distributed [ *N* (σ, µ; 0, 1)] as determined by Kolmogorow–Smirnow test for parametric variables and Shapiro–Wilk test for non-parametric variables. The mean values were compared by the least significant difference (LSD) option when interaction terms significant. The histopathology scores (discrete variables) were reported in median values after the Mann–Whitney U test (Version 13.2.2; MedCalc, Ostend, Belgium). Differences at p < 0.05 were considered statsistically significant.

## 3. Results

Table 1 shows inflammatory markers in response to the BA treatment upon liver injury induced by the APAP administration. Elevations in TLR-9 (p < 0.0037), IL-18 (p < 0.0007), NF-κB (p < 0.0001), and MDA (p < 0.0001) levels indicated accorrence of liver injury. The health status by the treatment interaction revealed that while the BA treatment did not change the inflammatory markers in the healthy rats, it was effective to reduce these inflammatory markers in the the APAP administrated rats. Increased serum concentrations of metabolites (exepcet for HDL, which increased) occurrence of liver injury in the APAP administered rats (Table 2; p < 0.0001 for all). The health status by the treatment interaction revealed that the BA administration was effective to reverse elevations in the serum concentrations in glucose (p < 0.0873), TChol (p < 0.0154), TG (p < 0.0002), LDL (p < 0.0012), AST (p < 0.0001) and upon the APAP administration, but not to the levels of the control group.

**Table 1:** Effect of the betulinic acid (BA) treatment on inflammatory markers on a liver injury-induced by the acetaminophen (Damaged) in rats.

		Parameters1			
Main groups and interaction		TLR-9pg/mL	IL-18pg/mL	NF-κBng/mL	MDAnmol/L
Health Status					
	Healthy	3.58 ± 0.18	0.67 ± 0.04	8.09 ± 0.50	4.94 ± 0.16
	Damaged	5.39 ± 0.73	0.96 ± 0.09	12.26 ± 1.15	8.19 ± 0.50
Treatment					
					
	None	5.80 ± 0.69	0.97 ± 0.09	12.4 ± 1.2	7.70 ± 0.64
	BA	3.22 ± 0.24	0.68 ± 0.05	8.17 ± 0.47	5.67 ± 0.25
Healthy					
	None	3.58 ± 0.25b	0.67 ± 0.05b	7.57 ± 0.75b	5.02 ± 0.25c
	BA	3.58 ± 0.26b	0.66 ± 0.05b	8.75 ± 0.56b	4.84 ± 0.20c
Damaged					
	None	7.80 ± 0.91a	1.23 ± 0.10a	16.8 ± 0.8a	10.1 ± 0.4a
	BA	2.97 ± 0.36b	0.70 ± 0.08b	7.76 ± 0.69b	6.26 ± 0.29b
Effect		p value			
Health Status		0.0037	0.0007	0.0001	0.0001
Treatment		0.0002	0.0017	0.0001	0.0001
Health Status x Treatment		0.0002	0.0027	0.0001	0.0001

1Data are the least square means + standard error of a mean, generated from two-way ANOVA. Different superscripts within columns differ in the interaction row (p < 0.05), attained by the least significant difference option in the post-Hoc test.

**Table 2:** Effect of the betulinic (BA) acid treatment on metabolic profile on a liver injury-induced by the acetaminophen (Damaged) in rats.

		Parameters1					
Main groups and interaction		Glucosemg/dL	TCholmg/dL	TGmg/dL	HDLmg/dL	LDLmg/dL	ASTU/L
Health Status							
	Healthy	105 ± 3	151 ± 5	104 ± 8	52.7 ± 1.8	77.3 ± 5.1	45.9 ± 3.0
	Damaged	74.0 ± 4.6	208 ± 9	184 ± 15	41.1 ± 4.1	131 ± 9	118 ± 13
Treatment							
	None	76.8 ± 5.6	190 ± 12	156 ± 21	38.4 ± 3.7	120 ± 12	110 ± 16
	BA	99.8 ± 3.8	174 ± 7	140 ± 6	55.1 ± 2.0	92.6 ± 5.8	60.1 ± 6.0
Healthy							
	None	97.4 ± 4.0b	146 ± 7c	82.5 ± 9.1c	53.7 ± 2.5b	75.9 ± 7.5c	45,6 ± 4,6c
	BA	114 ± 3a	157 ± 6c	132 ± 5b	51.4 ± 2.7b	79.1 ± 7.0c	46.4 ± 4.0c
Damaged							
	None	58.3 ± 4.9d	229 ± 14a	223 ± 23a	24.5 ± 1.8b	160 ± 10a	167 ± 12a
	BA	89.7 ± 3.4c	186 ± 10b	146 ± 10b	57.7 ± 2.6a	102 ± 7b	69.7 ± 8.6b
Effect		p value					
Health Status		0.0001	0.0001	0.0001	0.0001	0.0001	0.0001
Treatment		0.0001	0.1341	0.3868	0.0001	0.0032	0.0001
Health Status x Treatment		0.0873	0.0154	0.0002	0.0001	0.0012	0.0001

1Data are the least square means + standard error of a mean, generated from two-way ANOVA. Different superscripts within columns differ in the interaction row (p < 0.05), attained by the least significant difference option in the post-Hoc test.

**Figure 1:**
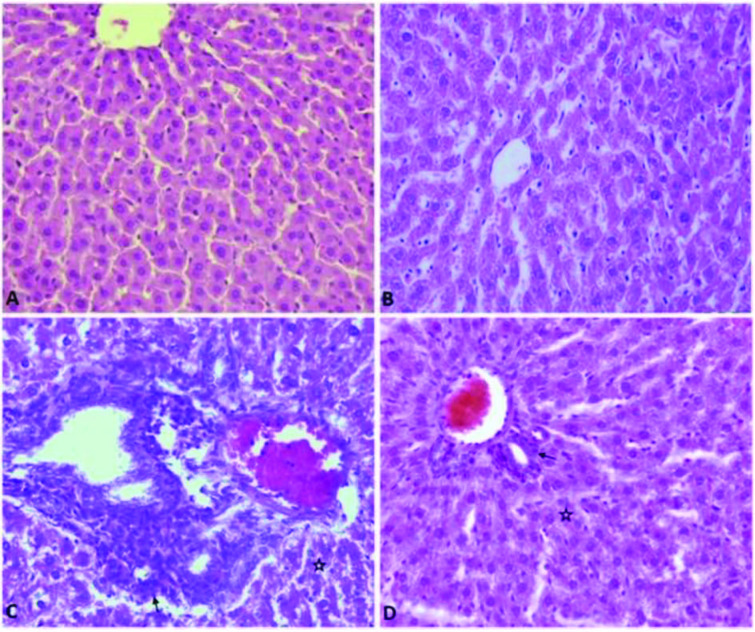
Effect of the betulinic (BA) acid treatment on histopathological evaluation of a liver injury-induced by the acetaminophen (APAP) in rats. A) No histopathological lesions in the control group (Group Control). Degenerative changes and necrosis in the hepatocytes (star), inflammation cells (arrow), B) Mild (BA group), C) Severe (group APAP), D) Moderate (APAP+BA), HXE, Bar: 40 μm.

No histopathological lesions were determined in liver tissues of the control group (Figure 1A). The APAP group showed congestion and dilatation in the liver blood vessels. Severe degeneration (vacuolar or hydrophic degeneration) and necrosis were seen in the hepatosites and hyperplasia of bile ducts. In addition to these changes, mononuclear cells had infiltrated in intralobuler and portal areas to these changes. Furthermore, fat droplets in some of hepatocytes were observed (Table 3). Although similar lesions were observed in the APAP + BA and BA groups, it was noted that the severity of these lesions was highest in the APAP group (Figures 1B, 1C, 1D).

**Table 3:** The semi-quantitative histopathological evalution of the hepatocytes in response to the betulinic acid (BA) treatment upon a liver injury-induced by the acetaminophen (Damaged) in rats.

				Parameters1			
Main groups and interaction		Degenerative necrosis	Inflammatory cells	Bilier hyperpathia	8-OHdG		IL-1β
Health Status							
	Healthy	0.5 (0–2)	0.0 (0–2)	0.0 (0–1)	0.5 (0–2)		0.5 (0–2)
	Damaged	3.0 (2–4)	2.5 (1–4)	2.0 (0–3)	2.0 (0–3)		3.0 (2–4)
Treatment							
	None	2.0 (0–4)	1.5 (0–4)	1.0 (0–3)	1.5 (0–3)		1.5 (0–4)
	BA	2.0 (0-)	1.0 (0–3)	1.0 (0–3)	1.0 (0–3)		2.0 (0–3)
Healthy							
	None	0.0 (0–0)d	0.0 (0–3)c	0.0 (0–3)b	0.5 (0–3)c		0.0 (0–4)d
	BA	1.0 (0–2)c	0.5 (0–3)c	0.5 (0–3)b	0.5 (0–3)c		1.0 (0–3)c
Damaged							
	None	3.5 (2–4)a	3.0 (1–4)a	2.5 (0–3)a	3.0 (2–3)a		3.0 (2–4)a
	BA	2.5 (2–4)b	2.0 (0–4)b	2.0 (0–3)a	1.5 (0–3)b		2.5 (0–4)b
Effect		p Value					
Health Status		0.0001	0.0001	0.0001		0.0001	0.0001
Treatment		0.7682	0.3795	0.4535		0.0018	0.5263
Health Status x Treatment		0.0001	0.0464	0.2156		0.0205	0.0001

1Data are the median (min–max) values, generated from the Mann Whitney U test. Different superscripts within columns differ in the interaction row (p < 0.05), attained by the ranking.

**Figure 2:**
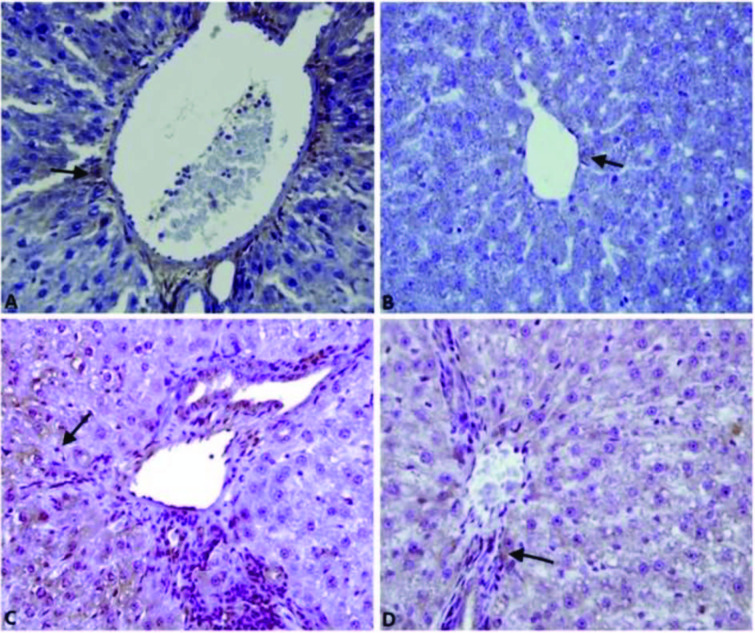
Effect of the betulinic (BA) acid treatment on immunohistochemstry of a liver injury-induced by the acetaminophen (APAP) in rats. Immunohistochemstry stain; immunopositivity for 8-OHdG in the hepatocytes (arow). A) Mild (Group Control), B) Mild (BA group), C) Severe (group APAP), D) Moderate (APAP+BA), Bar: 40 μm.

**Figure 3:**
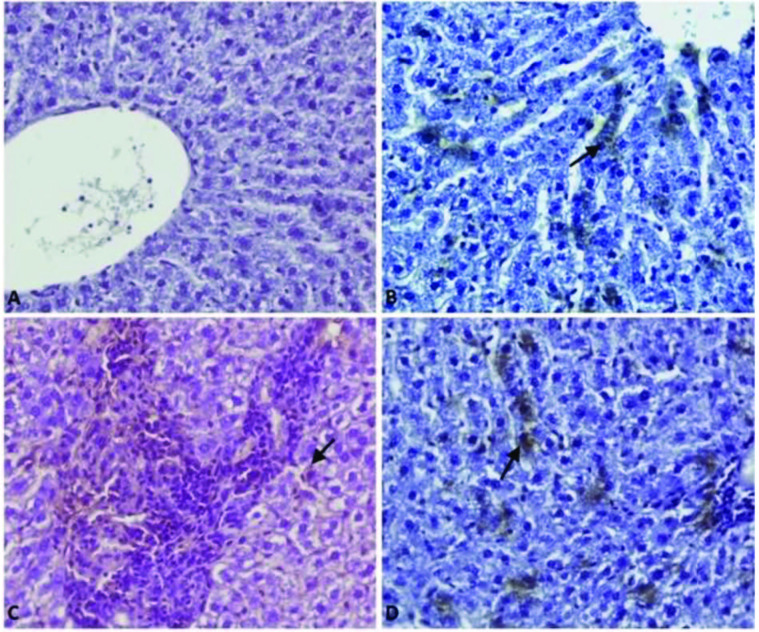
Effect of the betulinic (BA) acid treatment on immunohistochemstry of a liver injury-induced by the acetaminophen (APAP) in rats. Immunohistochemstry stain; immunopositivity for IL-1β in the hepatocytes (arow), A) Mild (Group Control), B) Mild (BA group), C) Severe (group APAP), D) Moderate (APAP+BA), Bar: 40 μm.

All groups showed immunopositivity for 8-OHdG and IL-1β in the hepatocytes, inflammatory cells, and epithelial cells of of bile ducts. The positive reaction of 8-OHdG (Figures 2A, 2B, 2C, 2D) and IL-1β (Figure 3A, 3B, 3C, 3D) was the highest in the APAP group and the least in the control group. 

## 4. Discussion

Liver, having important functions in drug metabolism, is the major target organ for drug toxicity. Hepatotoxicity due to drug damage is a common cause of acute liver damage [23]. It is highly important to elucidate the mechanisms of action of hepatotoxicity, posing a risk for mortality, for the biological follow-up of toxic substances and developing treatment methods. Acetaminophen causes lethal hepatocellular necrosis in experimental and clinical studies, whether it is used at therapeutic doses or at high doses [24].

Laboratory tests used in the diagnosis, follow-up and treatment of liver diseases indicate liver damage and functional status of the liver and they are called routine liver tests. The AST is one of the significant parameters of liver cell damage and a routine liver test assessing also the presence, character, and treatment response of liver toxicity. In our study, we observed that serum AST levels were increased by the APAP administration and decreased by the BA treatment, which are in agreement with previous studies [25,26]. Upon the APAP administration, catabolic profile existed, as reflected by increases in serum glucose, TChol, TG, and LDL levels and a decrease in serum HDL levels. The hpperlipidemic effect of the APAP administration may reflect the deterioration of liver cells to metabolize lipids or lipid peroxidation. The increase in serum lipids may be attributed to the increased liver synthesis and/or diminished liver degradation; reduced lipoprotein lipase activity plays a role in the lipids increment [27].

The liver itself is affected by the toxicities mostly, due to its metabolic functions, and endogenous antioxidant defense mechanisms fail and undesirable severe clinical conditions develop. Lipid peroxidation products increasing due to tissue damage in toxic hepatitis may interfere with various biomolecules at the site of damage and may interfere with the functions of these biomolecules [28]. Lipid peroxidation was also suggested to play an important role in the APAP-induced hepatotoxicity. Aktaş et al. [29] reported that both plasma and liver MDA levels increased in toxic hepatitis induced by the APAP administration, which is attributed to free radicals’ production caused by weakening of antioxidant system.

Various exogenous antioxidants are used to control and reduce increasing lipid peroxidation in living beings [30]. The BA administration significantly decreased plasma MDA, AST, TG, TChol, and LDL levels and increased HDL levels. In a study, Yi et al. [31] investigated the liver protective effects of BA in alcohol induced liver damage and reported that the BA treatment had hepatoprotective effects. In another study it was shown that the BA treatment was noted to significantly decrease plasma AST levels and inhibit apoptosis in the liver [32]. It appears that the hepatoprotective mechanism is linked to BA’s antioxidant capacity, mainly by improving the tissue redox system, maintaining the antioxidant system, and decreasing lipid peroxidation in the liver [33].

Activation of TLR increases the synthesis and release of molecules, and causes tissue damage with the emergence of free radicals, in various inflammatory events including increase of proinflammatory cytokine levels. The cytokines involved in the induction of immune response provide intercellular communication and play an effective role in influencing the severity and the maintenance of the immune response [34]. Imaeda et al. [35] reported that the APAP administration resulted in significant damage in the hepatocytes. However, they noted that free DNA released from apoptosis-induced hepatocytes activated TLR-9 levels, and the increased TLR-9 enhanced the transcription of IL-18 encoding genes. In another study conducted by Teratani et al. [36] in 2017, an increased TLR-9 level was interpreted as an important indicator of acute liver damage. They also reported that the severity of liver damage increased in line with the activation of TLR-9/inflammasome pathway. In this study the APAP administration increased TLR-9 and IL-18 levels, as well.

NF-κB is the major modulator of all TLR signaling mechanisms, and the activation of NF-κB plays a critical role in TLR-mediated activation of the innate immune response. Its rapid activation is crucial for the required immune response, because all TLR signaling pathways result in NF-κB activation, controlling the expression of a number of inflammatory cytokine genes [37,38]. In an experimental study, Zhang et al. [39] induced liver fibrosis by carbontetra chloride (CCl4) and noted that NF-κB and TLR-4 increased mRNA levels. The ROS-induced lipid peroxidation products such as lipid aldehydes in the activation of signaling cascade eventually activates NF-B, indicating that ROS-induced lipid peroxidation has been proposed to be majör contributor in the pathophysiology of many inflammatory disorders [40]. In this experiment, the APAP administration resulted in significant increase in TLR-9, NF-κB and IL-18 levels as well, accompanied by increases in MDA levels, in agreement with the literature [41]. Elevations in these molecular parameters were reversed by the BA treatment. There are not many studies in the literature investigating the effects of the BA treatment on liver damage. Liu et al. [42] stated that BA exerted hepatoprotective effects through autophagy, suggesting that it could be used as a new agent in the treatment of hepatic fibrosis. This beneficial effect is related to its high antioxidant capacity to suppress lipid peroxidation and activate the endogenous antioxidant system [43]. In an experimental study examining the effects of BA on renal fibrosis, it was reported that BA prevents fibrosis by inhibiting NF-κB activation [44]. The present findings reinforce the potential of BA, a natural compound as an anti-inflammatory drug candidate consider the role of TLR-9/NF-κB /IL-18 as another important mediator involved in the immune regulation produced by the APAP and indicate the carrying out of future clinical evaluations involving BA effect on severe liver diseases.

The hepatotoxicity of APAP reflected with sinusoidal dilatation, necrosis, and inflammatory cell infiltration in microscopic evaluation of liver tissue, which were evident in this experiment. The BA treatment is alleviated these adverse consequences of the APAP administration. 8-OHdG is another pathway to detect damages caused by ROS in the cell. 8-OHdG is known as an important indicator of DNA damage due to oxidative stress that can be induced by ROS and also a sign of cellular oxidative stress in DNA degradation [45]. IL-1β is one of the major mediators of chronic inflammatory diseases. Immunohistochemical analysis revelated that the APAP administration activated 8-OHdG and IL-1 β in liver tissue. It was observed that 8-OHdG and IL-1β staining was minimal in BA-treated rat livers.

## 5. Conclusion

In summary, APAP-induced acute liver damage is a serious clinical problem with high morbidity and mortality caused by oxidative stress and diffuse inflammation. Regarding pathophysiology of liver injury, TLR-9 had an important role in the initiation of hyperinflammation and development of tissue damage, which triggered NF-κB and IL-18 activation. The BA treatment inhibited TLR-9/NF-κB /IL-18 and lipid peroxidation for alleviation of tissue damage. Therefore, BA can be used as an effective agent in the prevention and treatment of acute liver diseases, due to its inhibitory properties in multiple pathways and its potent antioxidant effects.

## Informed consent

This study was carried out in the Atatürk University’s Experimental Animal Laboratory of the Medical and Experimental Application and Research (ATADEM) in accordance with the Atatürk University’s Local Ethical committee decision (2018/34).
